# Effect of Additives on CO_2_ Adsorption of Polyethylene Polyamine-Loaded MCM-41

**DOI:** 10.3390/molecules29051006

**Published:** 2024-02-26

**Authors:** Xia Wang, Wulan Zeng, Peidan Hu, Shengxin Liu, Yuechao Lin, Zhaowen He, Chunling Xin, Xiangjun Kong, Jinghan Xu

**Affiliations:** Department of Chemistry and Chemical Engineering, Weifang University, Weifang 261061, China; 20110786@wfu.edu.cn (W.Z.); 21071340014@wfu.edu.cn (P.H.); 21071340010@wfu.edu.cn (S.L.); 21071240004@wfu.edu.cn (Y.L.); 21071240009@wfu.edu.cn (Z.H.); 20150015@wfu.edu.cn (C.X.); 20170003@wfu.edu.cn (X.K.);

**Keywords:** coimpregnation, CO_2_ adsorption, chemisorption, additive, regeneration

## Abstract

Organic amine-modified mesoporous carriers are considered potential CO_2_ sorbents, in which the CO_2_ adsorption performance was limited by the agglomeration and volatility of liquid amines. In this study, four additives of ether compounds were separately coimpregnated with polyethylene polyamine (PEPA) into MCM-41 to prepare the composite chemisorbents for CO_2_ adsorption. The textural pore properties, surface functional groups and elemental contents of N for MCM-41 before and after functionalization were characterized; the effects of the type and amount of additives, adsorption temperature and influent velocity on CO_2_ adsorption were investigated; the amine efficiency was calculated; and the adsorption kinetics and regeneration for the optimized sorbent were studied. For 40 wt.% PEPA-loaded MCM-41, the CO_2_ adsorption capacity and amine efficiency at 60 °C were 1.34 mmol/g and 0.18 mol CO_2_/mol N, when the influent velocity of the simulated flue gas was 30 mL/min, which reached 1.81 mmol/g and 0.23 mol CO_2_/mol N after coimpregnating 10 wt.% of 2-propoxyethanol (1E). The maximum adsorption capacity of 2.16 mmol/g appeared when the influent velocity of the simulated flue gas was 20 mL/min. In addition, the additive of 1E improved the regeneration and kinetics of PEPA-loaded MCM-41, and the CO_2_ adsorption process showed multiple adsorption routes.

## 1. Introduction

Coal-fired power plants are considered the largest point sources of CO_2_ emissions around the world; thus, in these, the design and development of effective CO_2_ capture technologies have attracted great attention. Liquid amine absorption, membrane separation and solid adsorption methods have been widely studied to effectively capture CO_2_ from large CO_2_ emission points. Solid adsorption technology, especially the low-temperature sorbents of amine-loaded mesoporous supporting materials are considered the potential sorbents for CO_2_ capture from flue gases, due to their good adsorption performance at 50–70 °C, easy regeneration at 100 °C and weak equipment corrosion [1,2,3,4,5,6].

Two methods are usually adopted to prepare the solid amine chemisorbents. One is the grafting method, which loads aminosilanes or ionic liquids onto the pore surface of supporting materials by chemical reactions [7,8,9,10,11], and another is the wet impregnation method, which loads organic amines onto the pore surface of supporting materials by physical combinations [12,13,14,15,16,17,18,19,20,21,22,23,24,25,26,27,28,29]. The grafting method has the advantages of strong bonding between the supporting materials and aminosilanes and good regeneration performance but relatively fewer active sites, limiting the adsorption capacity of prepared composite solid sorbents; the impregnating method has the advantages of easy operation, less energy consumption and more active sites for CO_2_ adsorption, but the weak hydrogen bonding interactions between amines and supporting materials limit the cyclic regeneration performance. Liu et al. grafted alkyl-functionalized (3-Aminopropyl) triethoxysilane (APTES) onto the zeolite beta by a reflux reaction, and the prepared sorbent exhibited the adsorption capacity of 1.44 mmol/g and a high adsorption rate of t_1/2_ = 0.69 min, as well as good stability after 20 cycles [8]. Kishor et al. functionalized the three-dimensional cubic KIT-6 using (3-aminopropyl)triethoxysilane, *N*-[3-(trimethoxysilyl)propyl] ethylenediamine and *N*1-(3-trimethoxysilylpropyle)diethylene triamine by a post grafting method in the aqueous solution; the results showed that the water solution improved the grafting efficiency of aminosilanes, CO_2_/N_2_ selectivity and regeneration performance [9]. Zhao et al. prepared a series of polyethylenimine (PEI)-modified porous materials (MCM-41, SBA-15, KIT-6, mesoporous carbon/CMK-3, protonated titanate nanotube/PINT and fumed silica/FS) using the wet impregnation method, in which PEI-modified PTNT and FS showed better CO_2_ adsorption performance due to more abundant pores and better PEI dispersion [12]. Peng et al. prepared the mesoporous carbon from chitosan with a total pore volume of 0.304 cm^3^/g by a hard-template method, which was then impregnated with pentaethylenehexamine (PEHA) to adsorb CO_2_, and the highest CO_2_ adsorption capacity of 3.72 mmol/g was achieved under atmospheric pressure [14].

In spite of the relatively good adsorption performance for the grafted and impregnated porous sorbents, the low amine contents of aminosilanes and the agglomeration and weak volatility of liquid amines limited the adsorption capacity or amine efficiency. Thus, a combined method of either first grafting then impregnation was adopted [30,31,32], otherwise the additives were added during the impregnation of liquid amines into the supporting materials [33,34,35,36,37]. Sanz et al. synthesized the pore-expanded MCM-41-based sorbents, respectively, by grafting with aminosilane of aminopropyl orethylenediamino, impregnating with liquid amine of PEI or tetraethylenepentamine (TEPA), and a double functionalization method of first grafting with aminosilane then impregnating with organic amine; the results showed that their corresponding maximum CO_2_ adsorption capacity were respectively 1.75, 2.20 and 2.36 mmol/g, with amine efficiency separately being 0.38, 0.25 and 0.37 mol CO_2_/mol N, and a double functionalization method improved the dispersion and exposure of viscous amine [30]. In our previous study, the polyetheramine of poly(propylene glycol)bis(2-aminopropyl ether) (A) was chosen as the additive, and coimpregnated with TEPA into MCM-41 to prepare the composite CO_2_ sorbents [33]. Due to the high electron cloud density around the O atoms in A and good dispersion of TEPA in pore channels, the composites exhibited not only good physisorption at 30 °C and ideal chemisorption at 60 °C, but also high desorption efficiency; methoxypolyethylene glycol (MPEG) was codispersed and coimpregnated with PEPA to prepare composite sorbents for CO_2_ capture, in which the adsorption capacity and regeneration performance were improved with respect to that of PEPA-impregnated MCM-41 [35].

In this study, four ether compounds of 2-Propoxyethanol (1E), Butyl ether (2E), 2-Methoxyethyl ether (3E) and 1-Methoxy-2-propanol (4E) were selected as the additives and, respectively, coimpregnated with PEPA into MCM-41 to prepare the composite sorbents for CO_2_ capture from flue gases in coal-fired power plants. The effects of the mass ratio of PEPA to the ether, adsorption temperature and influent velocity on CO_2_ adsorption were investigated, the amine efficiency was calculated, and the regeneration and adsorption kinetics for the optimized sorbent were also further studied.

## 2. Results and Discussion

### 2.1. Characterization

**Figure 1:**
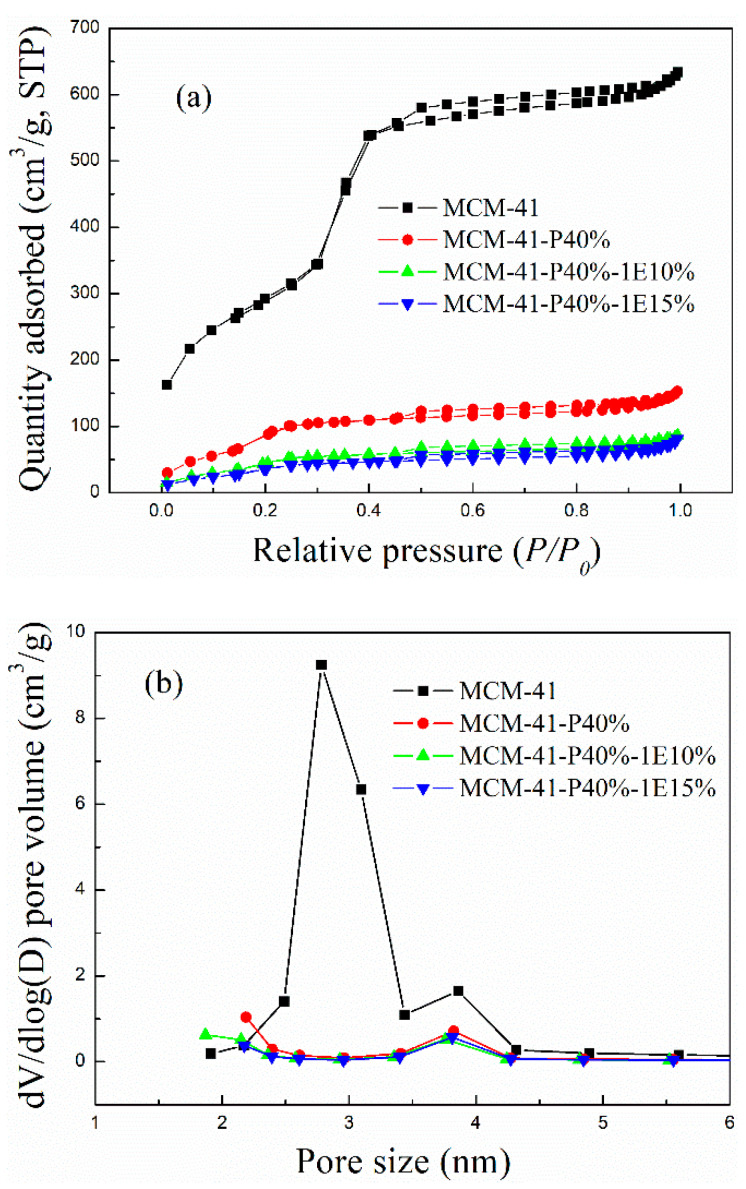
The (**a**) N_2_ adsorption–desorption isotherms and (**b**) pore size distribution curves for MCM-41 before and after PEPA, 1E impregnation.

**Figure 2:**
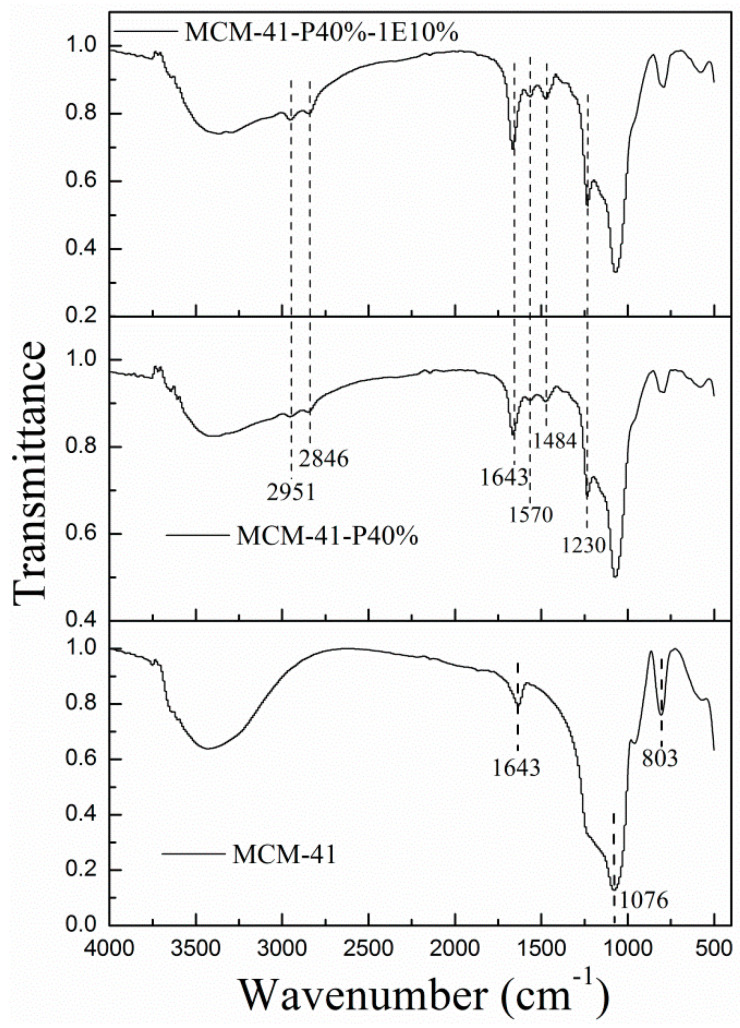
The FT-R spectra for MCM-1 before and after PEPA, 1E impregnation.

(1) N_2_ adsorption–desorption isotherms. The N_2_ adsorption–desorption isotherms, pore size distribution curves and pore structure data for MCM-41 before and after functionalization are shown in Figure 1a,b and Table 1. As shown in Figure 1a, the N_2_ adsorption for MCM-41 before and after PEPA and 1E functionalization exhibited type IV isotherms and H3 hysteresis loops when the relative pressure was above 0.3 and reversible pore filling steps when the relative pressure was below 0.3, indicating the mesoporous structure for the prepared composite sorbents. As can be seen from Figure 1b, after impregnating with PEPA and 1E, relatively small mesopores in MCM-41 were preferentially filled, showing that small mesopores suggested higher selectivity towards active composites, and PEPA and 1E had preferential entry into relatively small mesopores. As shown in Table 1, the specific surface area and pore volume gradually decreased as the load increased, suggesting that PEPA and the ether of 1E were loaded into the inner surface not the outer surface of MCM-41.

(2) FT-IR. Figure 2 shows the FT-IR spectra of MCM-41 before and after PEPA and 1E impregnation. For MCM-41 before and after functionalization, the asymmetric and symmetric stretching vibrations of Si–O–Si at 1076 and 803 cm^−1^ appeared, indicating that the skeletal structure for MCM-41 remained after impregnation. After 40 wt.% PEPA impregnation, the symmetric stretching vibrations and bending vibrations of N–H from the primary amine(–NH_2_) at 1484 and 1570 cm^−1^ appeared [32], the stretching vibrations of C–N from primary and secondary amine at 1230 cm^−1^ emerged [38], and the symmetric and asymmetric stretching vibrations of C–H in the alkyl groups at 2950 and 2845 cm^−1^ also appeared; compared with MCM-41, the peak for MCM-41-P40% at 1643 cm^−1^ was strengthened, which was not only from the bending vibrations of O–H in the adsorbed water, but also from the bending vibrations of N(R)H in PEPA. Above all, this suggested that PEPA was successfully loaded into MCM-41. Compared with MCM-41-P40%, the characterization peaks for MCM-41-P40%-1E10% all intensified, showing that the introduction of ether promoted the well dispersion and exposure of PEPA in MCM-41.

(3) Elemental analysis. Table 2 gives the elemental analysis results for MCM-41-P40% before and after 1E coimpregnation. In MCM-41-P40%, the elemental content of N was 10.54%, which increased, respectively, to 11.195% and 11.355% for MCM-41-P40%-1E10% and MCM-41-P40%-1E15%, suggesting that more N was loaded into the pores of MCM-41. The results were consistent with the FT-IR characterization. In this study, the ether was coimpregnated with PEPA to prepare the composite solid sorbents, in which the O atoms in the ether displayed hydrogen bonding or dipole interactions with PEPA and Si-OH in MCM-41, and the loading content of PEPA was improved than that of PEPA-impregnated MCM-41. Therefore, there was an insignificant increase in the nitrogen content.

### 2.2. The Effect of Additives on Adsorption and Amine Efficiency

**Figure 3:**
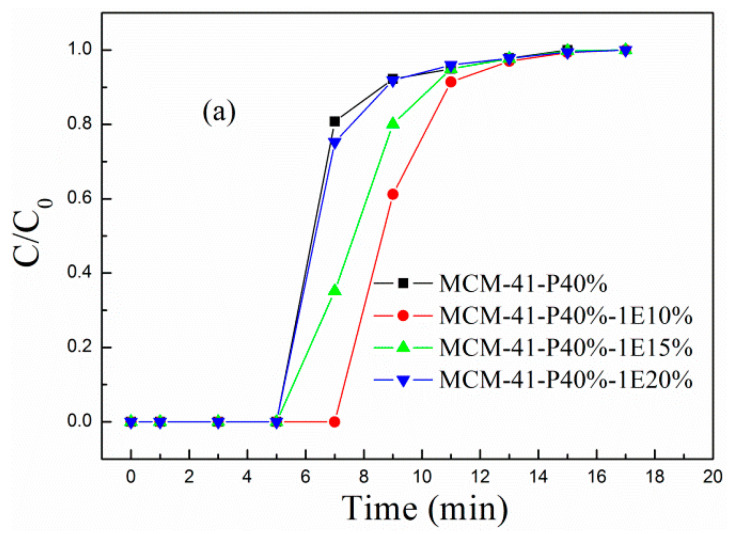

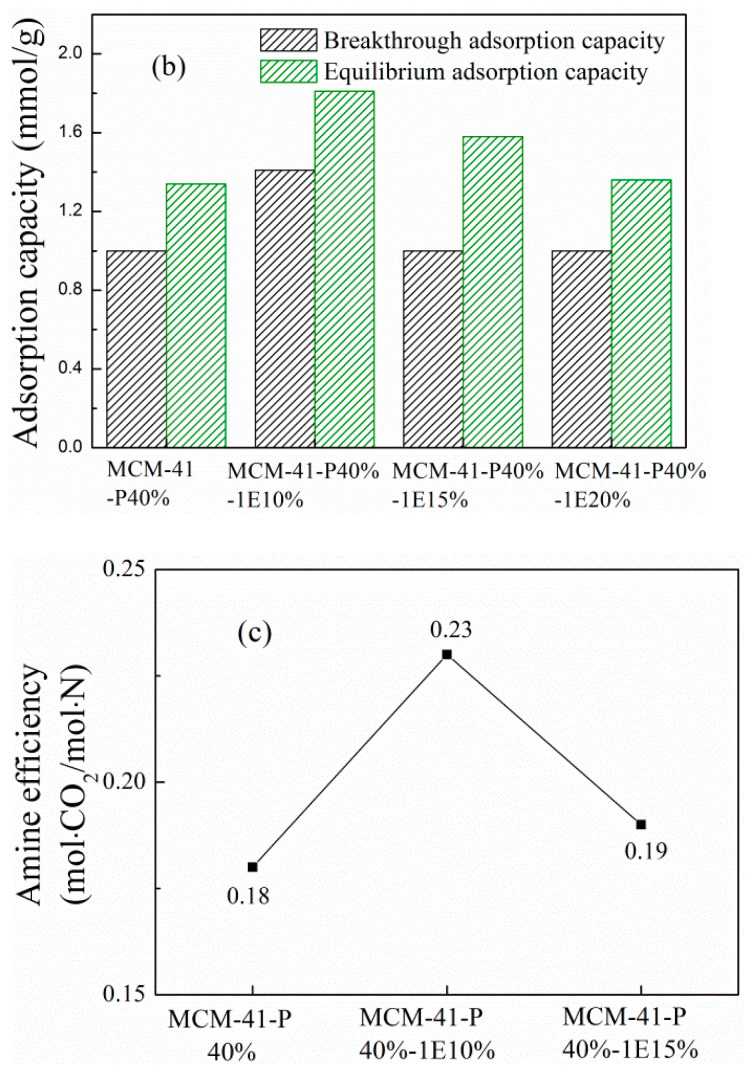
The (**a**) breakthrough adsorption curves, (**b**) adsorption capacity and (**c**) amine efficiency for MCM-41-P40% before and after 1E coimpregnation.

Figure 3 and Figure S1–S3 (in Supplementary Materials), respectively, describe the (a) breakthrough adsorption curves and (b) adsorption capacity for MCM-41-P40% before and after coimpregnation with 1E, 2E, 3E and 4E, when the influent velocity of the simulated flue gas was 30 mL/min and adsorption temperature was 60 °C. For MCM-41-P40%, the breakthrough time, breakthrough and equilibrium adsorption capacity were, respectively, 5 min, 1.00 and 1.34 mmol/g; as the 1E, 2E and 4E loading increased to 20 wt.%, the breakthrough adsorption curves all moved first to the right and then to the left, with corresponding breakthrough and equilibrium adsorption capacity first increasing and then decreasing; when the 1E loading was 10 wt.%, the breakthrough and equilibrium adsorption capacity were, respectively, 1.41 and 1.81 mmol/g, increasing 41% and 35.1%, respectively, compared to the corresponding value of MCM-41-P40%; when 2E loading was 10 wt.%, the breakthrough and equilibrium adsorption capacity were, respectively, 1.41 and 1.75 mmol/g, increasing to 41% and 30.6%, respectively, compared to corresponding value of MCM-41-P40%; when 4E loading was 10 wt.%, the breakthrough and equilibrium adsorption capacity were also, respectively, 1.41 and 1.75 mmol/g, increasing, respectively, to 41% and 30.6% compared to corresponding value of MCM-41-P40%. However, for the 3E-coimpregnated MCM-41, the breakthrough adsorption curves moved left, and corresponding adsorption performance decreased. Therefore, 1E, 2E and 4E-coimpregnation promoted the dispersion and exposure of the active sites in PEPA, in which the positive effect of 1E was the most obvious, so MCM-41-PEPA40%-1E10% was selected to further studied.

**Scheme 1:**
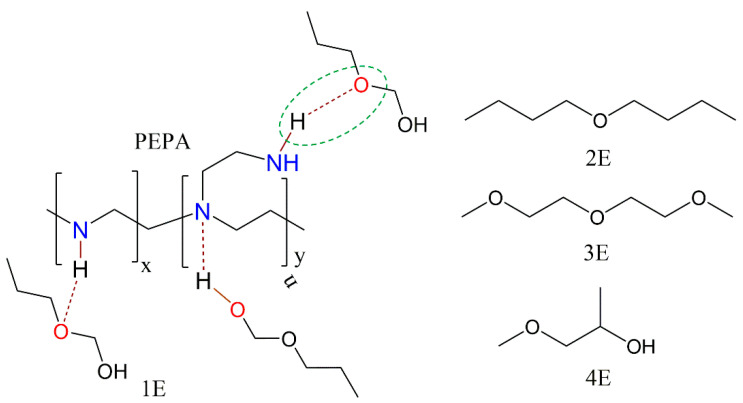
The chemical structures for PEPA and four ether compounds.

To further evaluate the effect of additives on CO_2_ adsorption, the amine efficiencies for PEPA-loaded MCM-41 before and after 1E coimpregnation were calculated; they are shown in Figure 3c. For MCM-41-P40%, the amine efficiency was 0.18 mol CO_2_/mol N; after 10% and 15% 1E coimpregnation, the amine efficiencies were 0.23 and 0.19 mol CO_2_/mol N, which, respectively, increased to 27.8% and 5.6% compared to the corresponding value of MCM-41-P40%. Therefore, the addition of 1E improved the amine efficiency of PEPA-loaded MCM-41. The ether and hydroxyl groups in 1E displayed hydrogen bonding interactions with the amino groups in PEPA (the chemical structures for PEPA and four ether compounds are shown in what follows (Scheme 1)), which promoted the dispersion of viscous PEPA in the pore channels of MCM-41; thus, more exposed active sites participated in CO_2_ capture. Similar phenomena were also reported by R. Sanz et al. [30] and X. Wang et al. [32].

Considering the good adsorption performance, MCM-41-P40%-1E10% was selected to be further studied.

### 2.3. The Effect of Adsorption Temperature

**Figure 4:**
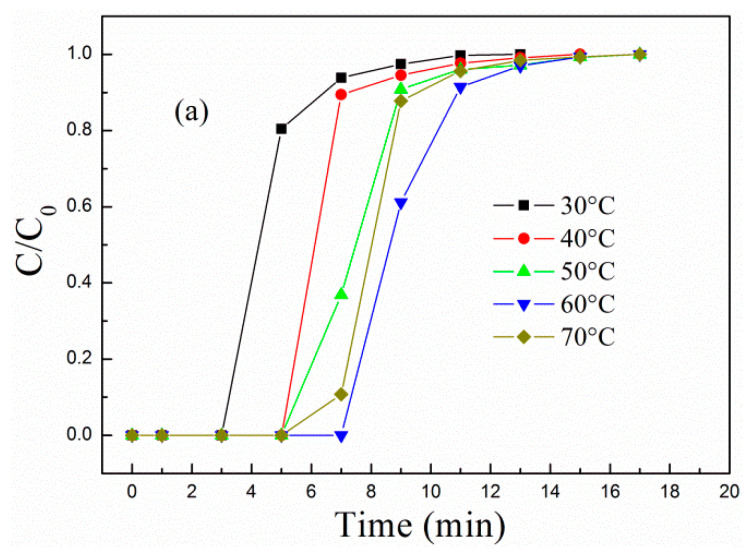

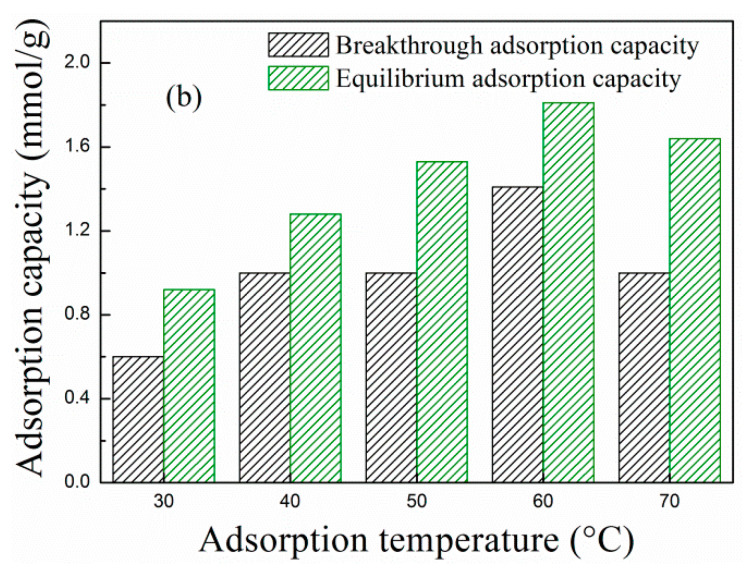
The (**a**) breakthrough adsorption curves and (**b**) adsorption capacity of MCM-41-P40%-1E10% at different adsorption temperatures.

Adsorption temperature is closely related to the adsorption type (physisorption or chemisorption). From a thermodynamic perspective, adsorption is exothermic, and low temperature is conducive to CO_2_ adsorption. However, PEPA is viscous, and high temperature favors the dispersion and exposure of viscous active components and promotes chemical reaction. Thus, further investigations are performed to select an optimal adsorption temperature. Figure 4 describes the (a) breakthrough adsorption curves and (b) adsorption capacity of MCM-41-P40%-1E10% at different adsorption temperatures when the influent velocity of the simulated flue gas was 30 mL/min.

As the adsorption temperature increased from 30 to 70 °C, the breakthrough adsorption curves in Figure 4a first moved to the right and then to the left, and corresponding breakthrough and equilibrium adsorption capacity in Figure 4b first increased and then decreased, suggesting that the adsorption of MCM-41-P40%-1E10% is mainly chemisorption. The higher the adsorption temperature, the more active the PEPA is, but adsorption is exothermic, and too high a temperature is not favorable for CO_2_ adsorption. Thus, the adsorption of MCM-41-P40%-1E10% first increased and then decreased as the adsorption temperature increased, and, at 60 °C, the breakthrough and equilibrium adsorption capacity separately reached the maximum value of 1.41 and 1.81 mmol/g.

### 2.4. The Effect of Influent Velocity

The influent velocity of the simulated flue gas relates to the collision probability of CO_2_ with active components. The higher the influent velocity, the more CO_2_ entering the reactor per unit time and the larger the collision probability of CO_2_ with active sites, so the sorbent will reach saturation in a shorter time.

**Figure 5:**
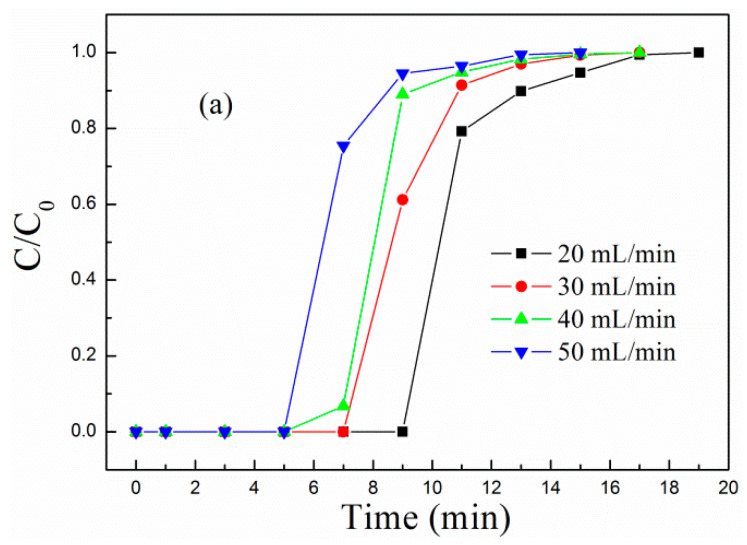

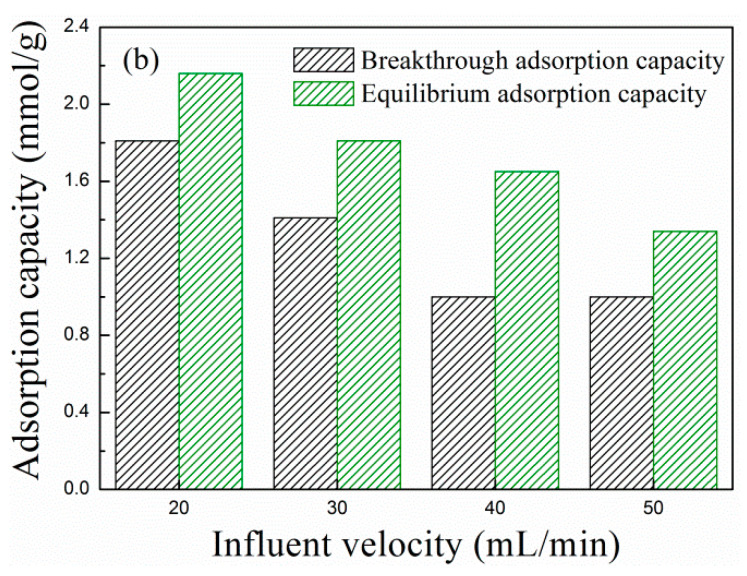
The (**a**) breakthrough adsorption curves and (**b**) adsorption capacity of MCM-41-P40%-1E10% at different influent velocities.

Figure 5 shows the (a) breakthrough adsorption curves and (b) adsorption capacity of MCM-41-P40%-1E10% at different influent velocities when the adsorption temperature was 60 °C. As the influent velocity increased from 20 to 50 mL/min, the breakthrough adsorption curves moved left, and the corresponding adsorption capacity decreased. When the influent velocity was 20 mL/min, the breakthrough and equilibrium adsorption capacity were, respectively, 1.81 and 2.16 mmol/g.

Based on the above, when the loading of PEPA and 1E were, respectively, 40% and 10%, and the adsorption temperature and influent velocity of the flue gas were 60 °C and 20 mL/min, the maximum equilibrium adsorption capacity of 2.16 mmol/g was reached, which was comparable with that of the related research. The adsorption data is shown in Table 3.

### 2.5. The Adsorption Kinetics of MCM-41-P40%-1E10%

**Figure 6:**
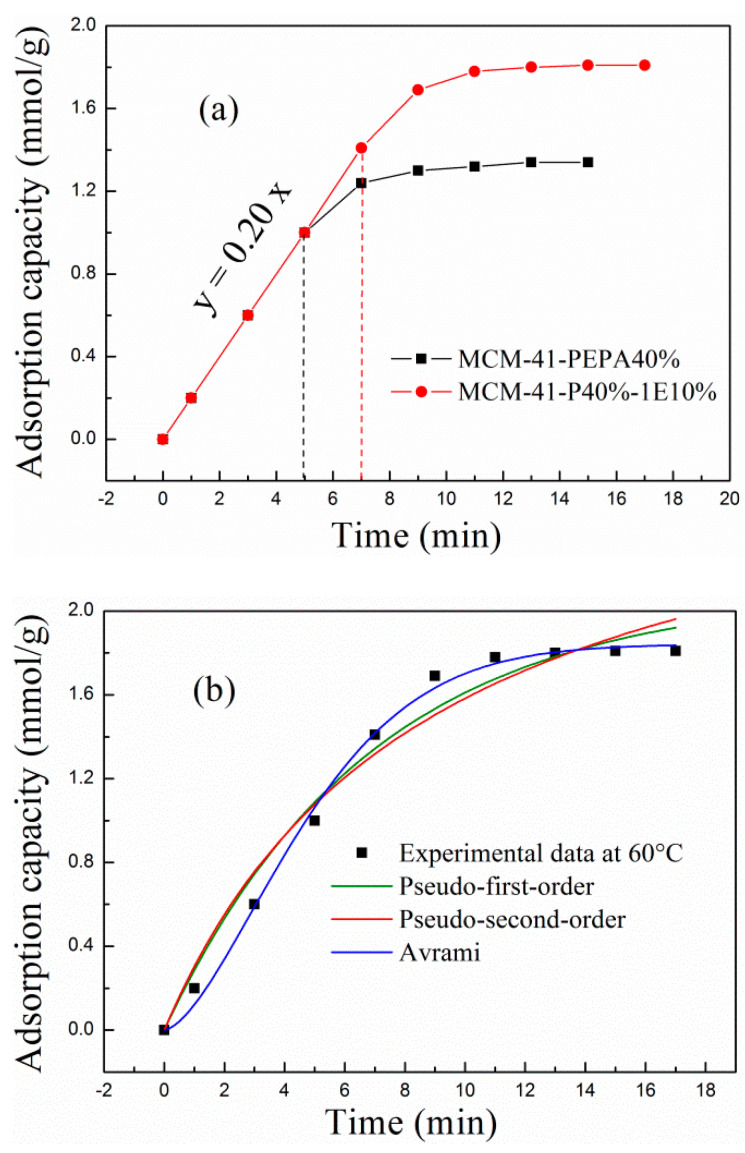
The (**a**) adsorption rate curves and (**b**) kinetic fitting of 1E−coimpregnated MCM-41-P40%.

The real-time adsorption capacity of MCM-41-P40% before and after 1E coimpregnation at 60 °C was calculated from corresponding breakthrough adsorption curves by the integration method; the adsorption rate curves (adsorption capacity versus time) are shown in Figure 6a. For MCM-41-P40% before and after 1E coimpregnation, the adsorption rates were kept at 0.20 mmol/g∙min before the breakthrough time, after which the adsorption rates rapidly decreased to a saturated adsorption condition. In particular, the breakthrough adsorption stage—that is, the rate constant stage for MCM-41-P40%-1E10%—was longer than that of MCM-41-P40%, suggesting that higher adsorption efficiency was reached for PEAP and 1E-coimpregnated MCM-41. The hydrogen bonding and dipole interactions among PEPA, ether and Si-OH in MCM-41 exposed more amino groups in PEPA, which participated in the reaction with CO_2_. Thus, 1E and PEPA-coimpregnated MCM-41 showed a longer breakthrough adsorption process.

To further investigate the adsorption kinetics of PEPA- and1E-coimpregnated MCM-41, the pseudo-first-order, pseudo-second-order and Avrami models were adopted to fit the adsorption data of MCM-41-P40%-1E10% at 60 °C; corresponding fitting curves and model parameters are shown in Figure 6b and Table 4. As shown in Figure 6b and Table 4, the adsorption data deviated from pseudo-first-order and pseudo-second-order curves but showed a good fit with the Avrami curve, with an R^2^ of 0.9956, suggesting that the Avrami model was more suitable for describing the adsorption process of MCM-41-P40%-1E10%. The adsorption process of MCM-41-P40%-1E10% was not simple physisorption or chemisorption, but involved multiple adsorption routes, and similar phenomena were also reported in other amine-modified mesoporous sorbents [39,40]. The physisorption was mainly due to the hydrogen bonding and dipole interaction among PEPA, ether and MCM-41, and chemisorption was mainly due to the chemical reaction between the active sites of amino groups in PEPA and CO_2_. The mechanism is shown in Equations (1)–(4).

CO_2_ + 2 RNH_2_ → RNHCOO^−^ + RNH_3_^+^ (1)

CO_2_ + 2 R_1_R_2_NH → R_1_NCOO^−^ + R_2_NH_2_^+^ (2)

CO_2_ + R_1_NH_2_ + R_2_NH → R_1_NH_3_^+^ + R_2_NCOO^−^ (3)

ROR′ + R_1_R_2_NH +CO_2_ → RR′OH + R_1_R_2_NCOO^−^ (4)

### 2.6. Regeneration

**Figure 7:**
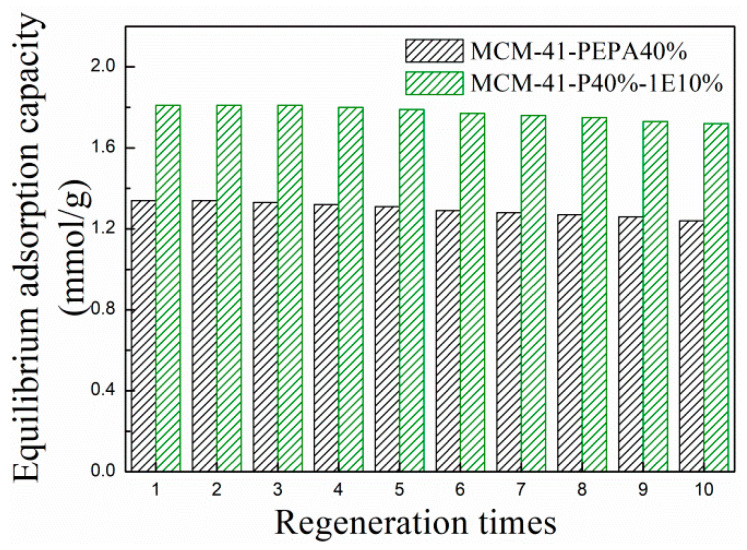
The equilibrium adsorption capacity for 1E-coimpregnated MCM-41-P40% after regeneration.

Good regeneration performance is an important index in evaluating a CO_2_ sorbent. Figure 7 describes the equilibrium adsorption capacity of MCM-41-P40%-1E10% during ten adsorption–desorption cycles, and, for comparison, corresponding data of MCM-41-P40% are also given. For MCM-41-P40%-1E10%, the equilibrium adsorption capacity during first three regenerations remained constant and then showed a slight decrease. After ten regenerations, the equilibrium adsorption capacity was 1.72 mmol/g, and was reduced by 5.0% compared to that of the fresh sorbent, which was lower than the corresponding data of MCM-41-P40% (7.5%). The coimpregnation of the ether introduced hydroxyl and ether groups in the pores of MCM-41, which displayed hydrogen bonding interactions with the amine groups from PEPA and promoted the dispersion and arrangement of easily agglomerated and volatile PEPA; thus, the regeneration performance for the ether and PEPA-coimpregnated MCM-41 improved compared to that of PEPA-loaded MCM-41.

## 3. Materials and Methods

### 3.1. Materials

PEPA and four ether compounds were bought from Shanghai Aladdin Bio-Chem Technology Co., Ltd. (Shanghai, China), MCM-41 was provided by Tianjin Nanhua Catalyst Co., Ltd. (Tianjin, China) and anhydrous ethanol was bought from Sinopharm Chemical Reagent Co., Ltd. (Shanghai, China). N_2_ (a purity of 99.999%), CO_2_ (a purity of 99.999%) and the simulated flue gas (15 vol.% CO_2_ diluted with N_2_) were provided by Weifang Weiyang Gas Co., Ltd. (Weifang, China).

### 3.2. Preparation of Coimpregnated Sorbents

The preparation of the composite sorbents adopted the wet impregnation method [33]. MCM-41 was ground and dried in a vacuum drying oven at 100 °C for 16 h. Certain volumes of PEPA and ether were transferred, respectively, into glass beakers, and 30 mL of anhydrous ethanol was added, which was then sonicated for 30 min. Afterwards, 1 g of MCM-41 was quickly added and the system was continuously sonicated for another 120 min and then transferred into the oven and dried for 24 h. The dried solid powder was labeled as MCM-41-P40%-aEb, in which a is 1, 2, 3 or 4 (the kind of the ether) and b is 10% or 15% (the weight loading percentage of the ether). Combing the pore structure characteristics, the loading percentage for PEPA was determined to be 40%.

### 3.3. Characterization

(1) Elemental analysis. The N element contents for MCM-41-PEPA40% before and after ether coimpregnation were performed on a CHNS Vario EL III elemental analyzer (Elementar, Germany). (2) FT-IR spectra. The surface functional groups for MCM-41 before and after PEPA and ether functionalization were tested using Fourier transform infrared spectroscopy via TENSOR-27 (Bruker, Germany); the frequency range is recorded over the range of 4000 to 500 cm^−1^. (3) N_2_ adsorption–desorption isotherms. The textural pore properties for MCM-41 before and after PEPA and ether modification were characterized on a Quadrasarb SI analyzer (Quantachrome, FL, USA). The BET surface area was obtained from the Brunauer–Emmett–Teller (BET) equation, the pore volume was determined from the N_2_ adsorption amount after completing capillary condensation at a relative pressure of 0.995, and the pore size distribution curves were drawn according to the desorption branch using the Barrett–Joyner–Halenda (BJH) model.

### 3.4. Adsorption and Regeneration

The adsorption and regeneration experiments were performed in a fixed bed reactor with an inner diameter of 0.8 cm and length of 40 cm. A total of 1.0 g of the pre-dried sorbent was first loaded in the center of the reactor and N_2_ was passed; the reactor was then heated to 100 °C and kept for 1 h to replace the air and moisture adsorbed in the sorbent, which was subsequently cooled to an adsorption temperature and the inlet gas was switched to the simulated flue gas. The outlet CO_2_ concentration of C was simultaneously collected by gas chromatography (GC). When C reached 5% of the inlet CO_2_ concentration of C_0_, the adsorption process finished breakthrough adsorption, during which the adsorption rate was rapid, and corresponding adsorption time and capacity were respectively breakthrough time and breakthrough adsorption capacity; when C was equal to C_0_, the adsorption reached saturation, and corresponding adsorption capacity was marked as equilibrium adsorption capacity. Both breakthrough and equilibrium adsorption capacities were calculated by integrating the breakthrough curves [32]. The integral equation of the breakthrough curve is displayed in Equation (5):

(5) $$Q_{s}=\frac{F\times \int_{0}^{t}\left(C_{0}-C\right)dt}{m}$$

where Q_s_ is the saturated adsorption capacity of CO_2_ over the sorbents, mmol·g^−1^; F represents the influent velocity of CO_2_, mL·min^−1^; m is the weight of the sorbent, g.

## 4. Conclusions

The coimpregnation of the ether additives (1E, 2E and 4E) with PEPA into MCM-41 refined the agglomeration and dispersion of viscous PEPA in MCM-41 and improved the CO_2_ adsorption capacity and amine efficiency as well as the regeneration performance of PEPA-loaded MCM-41. For MCM-41-P40% and MCM-41-P40%-1E10%, the equilibrium adsorption capacity and amine efficiency at 60 °C were, respectively, 1.34 mmol/g and 0.18 mol CO_2_/mol N, and 1.81 mmol/g and 0.23 mol CO_2_/mol N when the influent velocity was 30 mL/min, with the adsorption capacity and amine efficiency, respectively, increasing to 35.1% and 27.8%. When the loading of PEPA and 1E were, respectively, at 40% and 10%, and the adsorption temperature and influent velocity of the flue gas were 60 °C and 20 mL/min, the maximum equilibrium adsorption capacity of 2.16 mmol/g was reached. In addition, the regeneration and kinetics of 1E-coimpregnated PEPA-loaded MCM-41 also improved; the kinetic fit suggested that the adsorption process of coimpregnated MCM-41 was not simple physisorption or chemisorption, but involved multiple adsorption routes, where the hydrogen bonding and dipole interactions among PEPA, ether and MCM-41 contributed to physisorption, and the active sites of amino groups in PEPA contributed to chemisorption.

## Figures and Tables

**Table 1:** The pore structure data for MCM-41 before and after functionalization.

Sample	V_t_ (cm^3^/g)	S_BET_ (m^2^/g)	Pore Size (nm)
MCM-41	0.98	1078.15	3.08
MCM-41-P40%	0.24	405.19	3.42
MCM-41-P40%-1E10%	0.14	209.08	2.77
MCM-41-P40%-1E15%	0.12	165.53	3.86

**Table 2:** The elemental analysis results for MCM-41-P40% before and after 1E coimpregnation.

Sample	N (%)	C (%)	H (%)
MCM-41-P40%	10.54	18.5	5.369
MCM-41-P40%-1E10%	11.195	20.125	5.253
MCM-41-P40%-1E15%	11.355	20.555	5.22

**Table 3:** The equilibrium adsorption capacity of the solid sorbents both in the related study and this work.

Sorbent	Equilibrium Adsorption Capacity (mmol/g)	Adsorption Condition	Reference
APTES/zeolite	1.44	90 °C, 15% CO_2_	[8]
MCM-41-PEPA40%-MPEG10%	2.22	60 °C, 15% CO_2_	[35]
HMC-30TEPA	1.67	60 °C, 15% CO_2_ + 15% H_2_O	[39]
MCM-41-P40%-1E10%	2.16	60 °C, 15% CO_2_	This work

**Table 4:** The kinetic fit data for MCM-41-P40%-1E10% at 60 °C.

Kinetic Model	Parameter	60 °C
Pseudo-first-order	$q_{e}$ (mmol/g)	2.09
	$k_{1}$ (1/min))	0.1466
	R^2^	0.9776
Pseudo-second-order	$q_{e}$ (mmol/g)	2.98
	$k_{2}$ (g/mmol min)	0.0380
	R^2^	0.9685
Avrami	$q_{e}$ (mmol/g)	1.84
	$k_{a}$ (1/min)	0.1821
	$n_{a}$	1.58
	R^2^	0.9956

*q_e_*: the equilibrium adsorption capacity obtained from the fit curve; *k_1_*, *k_2_*, *k_a_*: the adsorption rate constants for each adsorption kinetic model.

## Data Availability

The data that support the findings of this study are available from the corresponding author, X. Wang, upon reasonable request.

## Supplementary Materials

The following supporting information can be downloaded at: https://www.mdpi.com/article/10.3390/molecules29051006/s1. Figure S1. The (a) breakthrough adsorption curves and (b) adsorption capacity for MCM-41-P40% before and after 2E-coimpregnation. Figure S2. The (a) breakthrough adsorption curves and (b) adsorption capacity for MCM-41-P40% before and after 3E-coimpregnation. Figure S3. The (a) breakthrough adsorption curves and (b) adsorption capacity for MCM-41-P40% before and after 4E-coimpregnation.
