# Supplementary material for: Effect of Additives on CO2 Adsorption of Polyethylene Polyamine-Loaded MCM-41
Source: Molecules. 2024 Feb 26;29(5):1006. doi: 10.3390/molecules29051006 (PMC10934004; doi:10.3390/molecules29051006)
Supplement: Supplementary file 1 [file molecules-29-01006-s001.zip › molecules-2872137-supplementary.pdf]

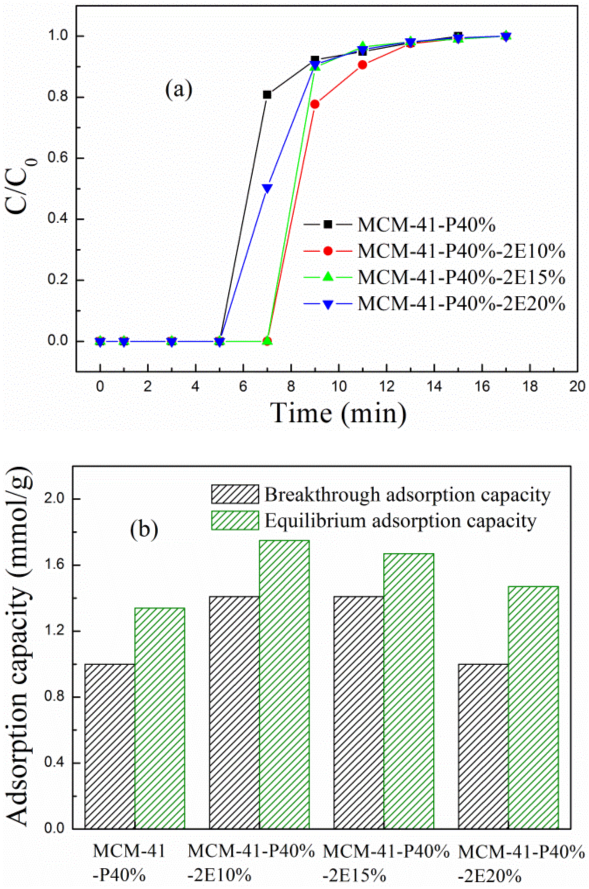


**Figure S1.** The (a) breakthrough adsorption curves and (b) adsorption capacity for MCM-41-P40% before and after 2E-coimpregnation.

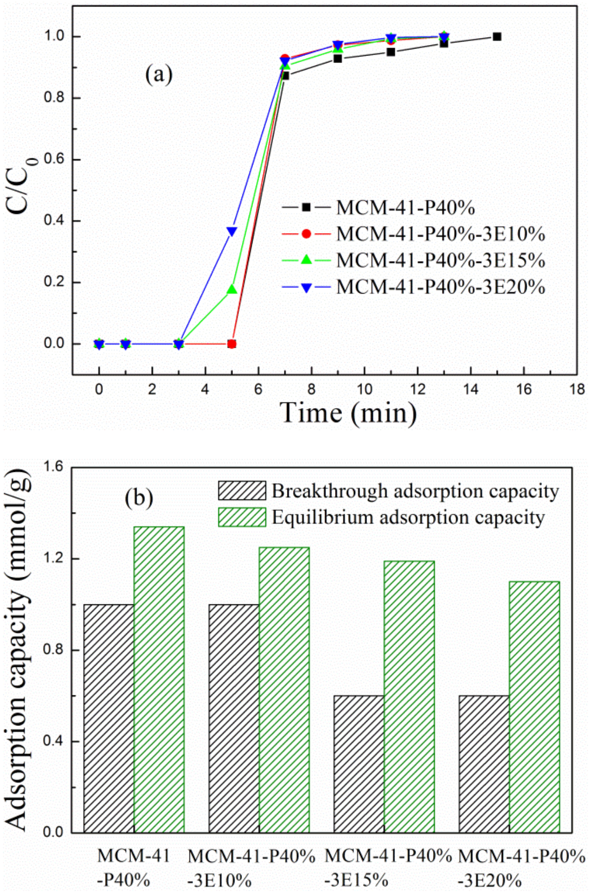


**Figure S2.** The (a) breakthrough adsorption curves and (b) adsorption capacity for MCM-41-P40% before and after 3E-coimpregnation.

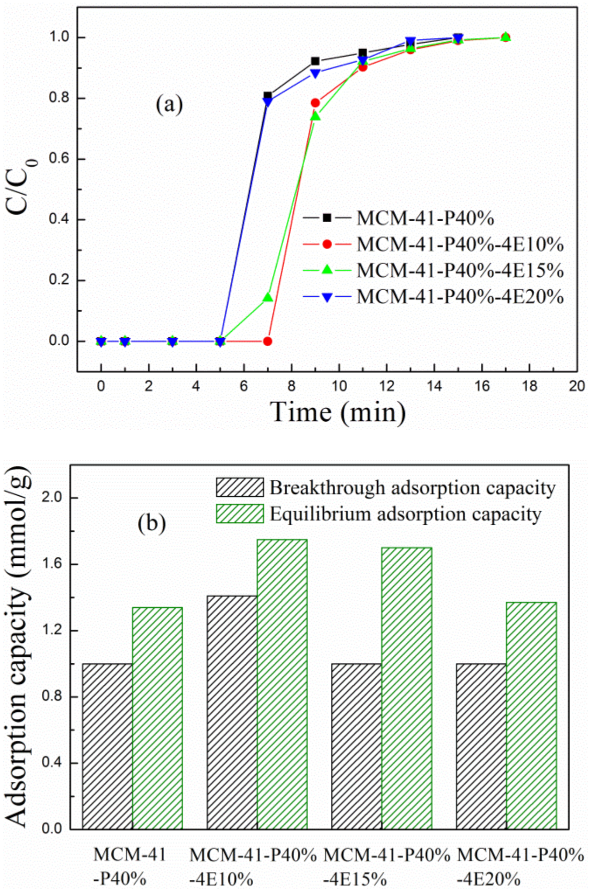


**Figure S3.** The (a) breakthrough adsorption curves and (b) adsorption capacity for MCM-41-P40% before and after 4E-coimpregnation.
